# Temporal expression analysis of angiogenesis-related genes in brain development

**DOI:** 10.1186/2045-824X-4-16

**Published:** 2012-10-01

**Authors:** Abdulkadir Özkan, Atilla Biçer, Timuçin Avşar, Askin Şeker, Zafer Orkun Toktaş, Süheyla Uyar Bozkurt, Ayse Nazli Başak, Türker Kılıç

**Affiliations:** 1 Marmara University, Institute of Neurological Sciences, Prof. Dr. Peter Black Laboratory of Molecular Neurosurgery, Istanbul, Turkey; 2 Boğaziçi University, Department of Molecular Biology and Genetics, Suna and Inan Kiraç Foundation, Neurodegeneration Research Laboratory, Istanbul, Turkey; 3 Istanbul Technical University, Dr. Orhan Öcal Giray Molecular Biology, Biotechnology and Genetics Research Center, Istanbul, Turkey; 4 Marmara University, Faculty of Medicine, Department of Neurosurgery, Istanbul, Turkey; 5 Marmara University, Institute of Neurological Sciences, Laboratory of Neuropathology, Istanbul, Turkey; 6 Bahcesehir University, Faculty of Medicine, Department of Neurosurgery, Istanbul, Turkey

**Keywords:** Cerebrovascular development, Arteriovenous malformations, Angiogenesis, Bai1

## Abstract

**Background:**

The current knowledge on molecular pathogenesis of cerebral vascular malformations (CVM), which are believed to arise during development, is very limited. To unravel the molecular mechanisms involved in CVMs, a detailed understanding of the brain vascular development at molecular level is crucial. In this study, we aimed to explore the temporal and comparative expression profile of angiogenesis-related genes in the establishment of brain vasculature.

**Methods:**

Expression of a total of 113 angiogenesis-related genes during murine brain development has been analyzed using low-density array systems designed for angiogenesis-related genes. Bai1 (brain specific angiogenesis inhibitor-1), a recently identified novel anti-angiogenic gene, has been selected for further characterization.

**Results:**

We found that 62 out of 113 analyzed genes have expression in brain development at varying levels. Nineteen of these were differentially expressed between embryonic and postnatal stages (>1.5 fold). Bai1 is strongly expressed on growing blood vessels of cerebral cortex and hippocampus, partially expressed in the lateral regions of striatum, but mostly absent on the thalamus.

**Conclusion:**

By showing the comparative expression analysis of angiogenesis-related genes throughout brain development, the data presented here will be a crucial addition to further functional studies on cerebrovascular research.

## Introduction

Embryonic vascular development is composed of a highly complex order of events that involve a variety of cell-cell interactions and a tightly balanced regulation of a wide range of functional molecules including growth factors and their receptors, transcription factors, cytokines, chemokines, proteases and their inhibitors, adhesion molecules and numerous matrix proteins. Actions of these factors must be well orchestrated in terms of time, space and dosage to form a functional vascular network [1-3].

In 1986 McCormick pathologically classified cerebrovascular malformations into the four following subgroups: Arteriovenous malformations (AVMs), cavernous malformations, venous angiomas and capillary telengiectasias [4]. Among these, AVMs are considered the most dangerous, with a high risk of intracranial hemorrhage [5]. AVMs are characterized with high-flow tangles of malformed arteries and veins without an intervening capillary bed. They bear a morphological resemblance to vascular plexus seen in development and are generally considered as congenital, resulting from defects in embryonic vascular specialization of the central nervous system [6]. Features of spontaneous hemorrhage, recurrence, growth and regression strongly suggest that AVMs are active lesions in terms of angiogenesis [7]. Moreover, a considerable amount of experimental studies demonstrated the presence of over-active, pathologic angiogenesis in AVMs, potentially being a reminiscent of embryonic development [8-14]. Our insights on the molecular biology of cerebral angiogenesis are decidedly limited to explain the causality of the AVM pathogenesis and to develop effective treatment modalities [8,15,16]. In this regard, elucidation of possible mechanisms and deciphering the molecular actors that orchestrate angiogenic genes in cerebral vascular development is essential.

In this study, we investigated the temporal and comparative expression of angiogenesis-related genes in mice brain development, using array systems specifically designed for angiogenesis.

## Methods

### Animal experiments

The study protocol was approved by the institutional Animal Care Committee of Marmara University, School of Medicine. Brain samples (Balb-c type) were obtained in different developmental stages between embryonic day 12 and postnatal day 20 and stored in liquid nitrogen.

### Array procedures

Low-density, pathway-specific membrane-array systems (OMM-024, SABiosciences) were used in the study. Each array contained 60-mer oligo probes for 113 genes previously indicated in angiogenesis, six housekeeping genes (Gapdh, Rps27a, B2m, Hspcb, Ppia as biological positive controls), six unrelated sequences and two probe-free empty spots (as negative controls) (Table 1). Brain samples belong to the embryonic E12, E14, E16, E18, E20, postnatal P1, P3, P5, P7, P9, P11, P13 days and adult animals have been used in the arrays. Homogenization, RNA isolation and cDNA synthesis were performed using MagNa-Lyser Homogenizer, High-Pure RNA Tissue and High-Fidelity cDNA Synthesis Kits (Roche) correspondingly. After assessment of the quality and quantity of the samples, complementary RNAs (cRNA) were synthesized by *in vitro* transcription (GA-030, SABiosciences) and labeled with biotin using biotinylated-UTP (Roche). cRNA samples obtained from 13 days were hybridized to the individual arrays and chemiluminescence was developed by alkaline phosphatase-conjugated streptavidin and CDP-star substrate system (SABiosciences). Image acquisition was done using Stella Image Acquisition System and Xstella 1.0 software (Raytest). Densitometric values were assigned by IDEA software (Image Data Extraction Applet, SABiosciences) and the data were analyzed by GEArray Expression Analysis Suite (SABiosciences). Assigned densitometric values were background corrected and normalized by the housekeeping genes Gapdh, Rps27a, Hsp90ab1, Ppia. The number of expressed genes was determined by IDEA software output and false-positives were removed after background correction and direct evaluation of captured raw array image by eye. Data were documented by clustergram and heat map graph and differentially expressed genes between em-bryonic and postnatal stages were determined by Mann–Whitney-U test (SPSS 17.0) and represented in scatter plot.

**Table 1:** List of genes found on the arrays

Gapdh*	**Adra2b**	**Angpt1**	**Angpt2**	**Akt1**	**Angptl3**	**Angptl4**	**Anpep**
Bai1	Ccl11	Ccl2	Cdh5	Col18a1	Col4a3	Csf3	Ctgf
Cxcl1	Cxcl10	Cxcl11	Cxcl2	Cxcl5	Cxcl9	Ecgf1	Edg1
Efna1	Efna2	Efna3	Efnb2	Egf	Eng	Epas1	Ephb4
Ereg	F2	Fgf1	Fgf2	Fgf6	Fgfr3	Figf	Flt1
Fzd5	Gna13	Hand2	Hgf	Hif1a	Ifna1	Ifng	Igf1
Il10	Il12a	Il18	Il1b	Il6	Itgav	Itgb3	Jag1
Kdr	Lama5	Lect1	Lep	Mapk14	Mdk	Mmp19	Mmp2
Mmp9	Notch4	Nppb	Npr1	Nrp1	Nrp2	Nudt6	Pdgfa
Pdgfb	Pecam1	Pgf	Plau	Plg	Plxdc1	Pofut1	Prok2
Pten	Ptgs1	Ptgs2	Ptn	Serpinf1	Sh2d2a	Smad5	Sphk1
Stab1	Stab2	Tbx1	Tbx4	Tek	Tgfa	Tgfb1	Tgfb2
Tgfb3	Tgfbr1	Thbs1	Thbs2	Timp1	Timp2	Timp3	Tmprss6
Tnf	Tnfaip2	Tnfrsf12a	Tnfsf12	Tnfsf15	Tnnt1	Vegfa	Vegfb
Vegfc	Wasf2	PUC18**	Blank**	Blank**	AS1R2**	AS1R1**	AS1**
Rps27a*	B2m*	Hspcb*	Hspcb*	Ppia*	Ppia*	BAS2C***	BAS2C***

* House-keeping gene.

** Unrelated probe and probe-free negative controls.

*** Biotin-conjugated positive controls.

### Confirmation of array data by qPCR

To confirm the array data, three genes; brain specific angiogenesis inhibitor-1 (Bai1), nudix (nucleoside diphosphate linked moiety X)-type motif 6 (Nudt6), and Natriuretic peptide receptor-1 (Npr1) were selected based on their novel with regard to their possible role in the development of brain and brain vasculature, and analyzed with quantitative real-time PCR (qPCR). qPCR was carried out by UPL system (LightCycler 2.0, Roche) with following conditions: initial denaturation at 95°C for 10 min, 45 cycles of denaturation at 95°C for 10 sec, annealing at 60°C for 30 sec, extension at 72°C for 1 sec, and final cooling at 40°C for 30 sec. Those reactions with error value outside the range of 0 ± 0.2 and efficiency values outside the range of 2 ± 0.5 were repeated and data was normalized to reference gene (Gapdh). Experiments were repeated three times using different brain samples. Primers and probes for Bai1 F: GGCCAAGAAT GAGAACGTG, R: CCAGTTCTGCATACCGTGATT, UPL probe#50: TCTGGAGC, for Nudt6 F:GACTCTG TGGCTGGGAGAAG, R: TCCTGGTGCTAACATCAA ATACA, UPL probe#3: GACCCAG, for Npr1 F: TGGA GACACAGTCAACACAGC, R: CGAAGACAAGTGGA TCCTGAG, UPL probe #60: TGGGGAAG.

### Immunohistochemistry

Tissue level expression of Bai1 was investigated in 4% paraformaldehyde-fixed brain samples belonging to days E15, P2 and P14 by the standard immunostaining procedure. Primary antibodies used in the study were; Bai1 (sc-66815, SantaCruz), CD31 (550274, BD Biosciences), PDGFR-β (AF1042, R&D Systems), secondary antibodies; donkey anti-goat conjugated with Alexa Fluor 488 (Invitrogen, A-11055), donkey anti-rabbit conjugated with Alexa Fluor 555 (Invitrogen, A-31572), donkey anti-rat, Cy5 (Millipore, AP189S).

## Results and discussion

As previously suggested by Mullan *et al.,* cerebrovascular malformations, AVMs in particular, are thought to arise during the establishment of brain vasculature [17]. Molecular mechanisms involved in the emergence of AVMs are far from being elucidated and no more than a few hypotheses exist [15,18,19]. It is known that these lesions are angiogenically active and may result from disruption of the fine balance between pro-angiogenic and anti-angiogenic molecules and/or defects during arterio-venous specialization of blood vessels during vascular development [10,20-23]. To clarify which molecular factors are impaired during AVM pathogenesis, norms of cerebrovascular development should be understood at molecular level. In the present study, we explored the expression dynamics of angiogenesis-related genes which play a role in normal cerebrovascular development, as an effort to contribute to the present level understanding of cerebral angiogenesis and AVM pathogenesis. We used low-density, pathway-specific array systems, which are designed to assess specific biological pathways. Therefore, compared to standard microarray chip systems, they provide added focus and more interpretable gene expression data.

### Temporal expression analysis revealed angiogenesis-related genes playing role in cerebrovascular development

**Figure 1:**
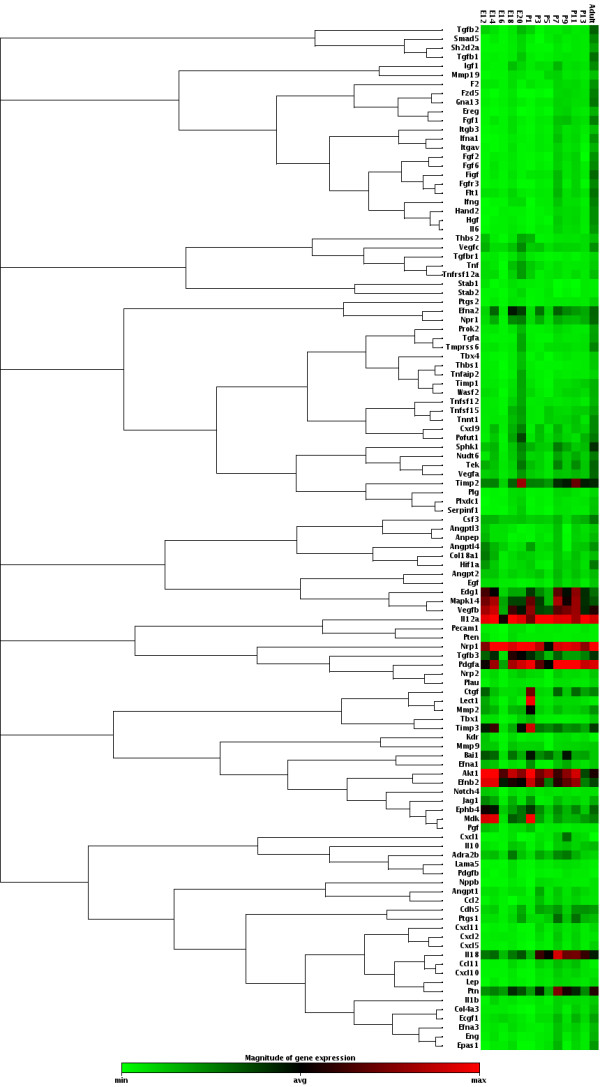
**Clustergram analysis and heatmap graph of gene expression data.** Developmental expression level of 113 angiogenesis-related genes was shown as heatmap graph and genes were clustered according to their expression patterns.

Our array analysis showed that 62 of 113 angiogenesis-related genes are expressed at varying levels during murine brain development (Figure 1, Table 2, Additional file 1: Table S1). The remaining 51 genes are either not expressed at all or expressed by a specific cell population at very minute levels, thus not detected in the array system. We have selected three novel genes, Bai1, Nudt6, and Npr1, to confirm the array data by qPCR analysis and observed similar expression patterns between array and qPCR (Additional file 2: Figure S1). Among these genes, Nudt6 is highly novel and there is no study pinpointing to its expression in any developmental process or stage. Nudt6 is synthesized from the complementary strand of Fgf2, and was implicated to function in post-transcriptional regulation of Fgf2 [24]. Studies on Nudt6 are very limited, and its roles are still unknown [25]. This study demonstrates the expression of Nudt6 during brain development for the first time, and its relatively high expression implies that it might have various crucial roles during CNS development (Table 2).

**Table 2:** Normalized expression values of 62 expressed genes according to functional groups

**Growth factors and receptors**					**Proteases, inhibitors and other matrix proteins**				
**Gene**	**E**	**P**	**Av**	**Adult**	**Gene**	**E**	**P**	**Av**	**Adult**
Bai1	0,24	0,27	0,26	0,05	Angptl4	0,12	0,09	0,10	0,11
Col18a1	0,13	0,08	0,10	0,07	Ecgf1	0,06	0,08	0,07	0,09
Ctgf	0,13	0,25	0,20	0,13	F2	0,04	0,05	0,05	0,11
Edg1	0,32	0,44	0,39	0,15	Mmp2	0,13	0,16	0,15	0,11
Egf	0,05	0,06	0,05	0,06	Mmp9	0,09	0,07	0,08	0,05
Ephb4	0,33	0,20	0,25	0,16	Plau	0,04	0,04	0,04	0,04
Fgf1	0,04	0,06	0,05	0,13	Timp2	0,34	0,38	0,36	0,22
Figf	0,03	0,06	0,05	0,16	Timp3	0,36	0,33	0,34	0,20
Flt1	0,05	0,06	0,06	0,15					
Igf1	0,06	0,06	0,06	0,12	**Transcription factors and others**				
Il18	0,27	0,59	0,45	0,23	**Gene**	**E**	**P**	**Av**	**Adult**
Jag1	0,14	0,09	0,11	0,08	Akt1	0,89	0,74	0,80	0,29
Kdr	0,06	0,04	0,05	0,01	Angpt2	0,09	0,09	0,09	0,07
Mdk	0,53	0,27	0,38	0,13	Efna1	0,08	0,09	0,09	0,05
Notch4	0,07	0,06	0,06	0,03	Efna2	0,26	0,19	0,22	0,16
Npr1	0,15	0,12	0,13	0,15	Efna3	0,05	0,06	0,05	0,07
Nrp1	0,96	0,83	0,88	0,52	Efnb2	0,70	0,58	0,63	0,19
Nrp2	0,07	0,06	0,06	0,06	Hif1a	0,10	0,07	0,08	0,14
Pdgfa	0,67	0,85	0,77	0,48	Lect1	0,08	0,19	0,15	0,02
Pgf	0,08	0,05	0,06	0,02	Mapk14	0,52	0,53	0,53	0,17
Prok2	0,06	0,04	0,05	0,09	Notch4	0,07	0,06	0,06	0,03
Ptn	0,29	0,38	0,35	0,30	Nudt6	0,11	0,10	0,11	0,14
Tek	0,14	0,13	0,13	0,16	Pofut1	0,14	0,09	0,11	0,14
Tgfb2	0,07	0,04	0,05	0,16	Pten	0,04	0,03	0,04	0,02
Tgfb3	0,39	0,25	0,31	0,21	Ptgs1	0,08	0,15	0,12	0,07
Tgfbr1	0,05	0,03	0,04	0,04	Sphk1	0,13	0,14	0,14	0,21
Tnfrsf12a	0,08	0,05	0,06	0,07	Tbx1	0,05	0,04	0,05	0,05
Vegfa	0,10	0,10	0,10	0,15					
Vegfb	0,62	0,58	0,60	0,29	**Cytokines and chemokines**				
Vegfc	0,13	0,08	0,10	0,11	**Gene**	**E**	**P**	**Av**	**Adult**
					Ccl2	0,03	0,07	0,05	0,03
**Adhesion molecules**					Csf3	0,13	0,11	0,12	0,14
**Gene**	**E**	**P**	**Av**	**Adult**	Cxcl9	0,12	0,09	0,10	0,12
Cdh5	0,09	0,15	0,12	0,10	Il10	0,06	0,07	0,07	0,07
Col18a1	0,13	0,08	0,10	0,07	Il12a	0,92	0,90	0,91	0,48
Ctgf	0,13	0,25	0,20	0,13	Tnf	0,08	0,05	0,07	0,05
Il18	0,27	0,59	0,45	0,23					
Nrp2	0,07	0,06	0,06	0,06					
Pecam1	0,03	0,03	0,03	0,02					
Thbs2	0,08	0,06	0,07	0,06					
Tnfrsf12a	0,08	0,05	0,06	0,07					

*Some genes are listed in more than one functional group. *E*: Embryonic average, *P*: Postnatal average, *Av*: Developmental average.

Comparative analysis of the array data revealed remarkable results: Between the three isoforms of widely known angiogenic stimulators, Vegf-A, -B and -C, Vegf-B had a 5-fold higher expression than the other two (Table 2), indicating it might have pivotal roles in neurodevelopment in addition to vascular development. Likewise, Nrp1, a co-receptor for Vegf-B and an axon guidance molecule of the semaphorin protein family, was expressed ten-fold more than the other two specific Vegf receptors, Flt1 and Kdr (Table 2). Also, between angiopoietin molecules (Ang1 and Ang2), which play significant roles in endothelial cell lumen stability [1], we found that Ang2 expression was equal to Vegf-A and Vegf-C, but its antagonist Ang1 seem to have no role during brain development (Table 2). Another notable finding is the Ephrin family member, Efnb2, which was 3–10 times more expressed than the other three ephrin ligands, Efna1, 2, 3, throughout brain development (Table 2). Similar comparisons are also possible for other major angiogenic molecules (like Tgf family members, Interleukins, Mmp family etc.) (Table 2). Such comparative analysis, (i.e. to be able to see the expression of all members of the same or close gene families at the same set) might be useful for further functional studies.

### Differentially expressed genes between embryonic and postnatal stages

**Figure 2:**
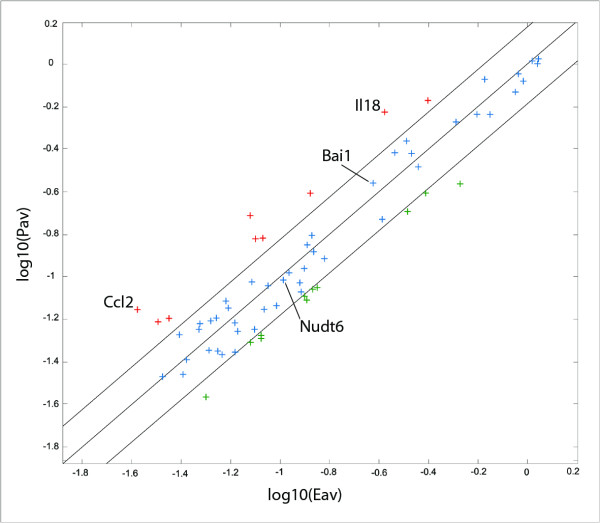
**Scatter plot analysis of array results shows the differentially expressed genes between embryonic and postnatal stages.** Graph denotes the logarithmic values of average gene expression in either stage. A total of 11 genes, Tgfbr4, Pgf, Tnf, Tnfrsf12a, Vegfc, Col18a1, Jag1, Pofut1, Ephb4, Tgfb3, and Mdk displayed higher expression in embryonic stage (shown in green). Eight genes, Ccl2, Figf, Fgf1, Lect1, Ptgs1, Cdh5, Ctgf, and Il18 showed a higher level of expression at postnatal stage (shown in red), but with a significant difference for only Ccl2 (p: 0.011) and Il18 (0.028) between two phases. House-keeping genes were equally expressed in both stages, except for the B2m. Boundary value was assigned as 1.5 fold.

Among the 62 genes that were expressed, 19 displayed more than 1.5 fold higher expression in either stage of the development (Figure 2). Eleven genes [Tgfbr1 (Eav/Pav:1.84), Pgf (1.54), Tnf (1.59), Tnfrsf12a (1.65), Vegfc (1.55), Col18a1 (1.65), Jag1 (1.55), Pofut1 (1.57), Ephb4 (1.60), Tgfb3 (1.55), and Mdk (1.94)] were expressed higher in embryonic stage and eight genes [Ccl2 (Pav/Eav:2.64), Figf (1.91), Fgf1 (1.79), Lect1 (2.57), Ptgs1 (1.88), Cdh5 (1.78), Ctgf (1.88), Il18 (2.23)] displayed higher levels of expression in postnatal stage. Among these, only Ccl2 and Il18 have reached to a statistically significant difference (p: 0.011 and 0.028, respectively).

Ccl2 (chemokine (C-C motif) ligand 2) has been previously identified as a small cytokine involved in several inflammatory processes, but later it has been proposed to have functions in modulation of neuronal activity and neuroendocrine signaling and it was constitutively expressed by astrocytes [26,27]. Astrocytes begin to arise at late embryonic stages and populate the cortex largely within the first three weeks of postnatal development [28]. We have found that Cc12 expression increases 2.64-fold during postnatal development (Table 2, Figure 2) overlapping with astrocyte production, which indicates that neuromodulatory function of astrocytes might be mediated through the Ccl2.

Another significant molecule is Il18, previously not implicated in brain development. We have found that Il18 is strongly expressed throughout development with two-fold more expression in postnatal stage (Figure 2, Table 2). This data indicate that, besides its known function as a pro-inflammatory molecule [29], Il18 seem to have important roles in neuronal and vascular development; further functional studies may give new insights into the biology of this molecule. It might be also worthwhile to investigate the tissue level expression of Il18 in cerebrovascular malformations, since Il18 was shown to have a role in pathological angiogenesis in other diseases, like rheumatoid arthritis [30,31]. Such studies may be especially valuable, since Il18 can be used as a prognostic or clinical marker for cerebrovascular malformations and may be assessed through the analysis of cerebrospinal fluid.

### Bai1 is expressed on growing cerebral blood vessels

Bai1 is a recently identified G-protein coupled receptor that has been implicated to have a role in a few physiological and pathological processes. It has been originally discovered as an anti-angiogenic molecule acting under the regulation of p53 in an *in vivo* experimental angiogenesis model [32]. Later, this function was shown to be mediated, at least partially, through its cleaved products Vasculostatin-40 (Vstat40) and Vasculostatin-120 (Vstat120), which act as anti-angiogenic paracrine factors on endothelial cells, both *in vitro* and in tumor xenografts [33-36]. Bai1 was suppressed or mutated in several cancers and its lack of expression was correlated with poor clinical outcome [37-40]. Bai1 was also proven to function in engulfment of apoptotic cells by macrophages via recognizing the phoshatidylserine residues on apoptotic cells and activating the intracellular cascades for cytoskeletal remodeling and related pathways [41].

In adult mice brain, Bai1 is expressed by astrocytes, by almost all neurons and low levels by macrophages [42,43]. Based on its regulatory domains and interacting partners, Bai1 was hypothesized to have roles in cell adhesion, growth cone guidance, neurotransmitter release and potentially in some signal transduction pathways [33], however, there is no direct evidence regarding its biological functions in neural tissue.

**Figure 3:**
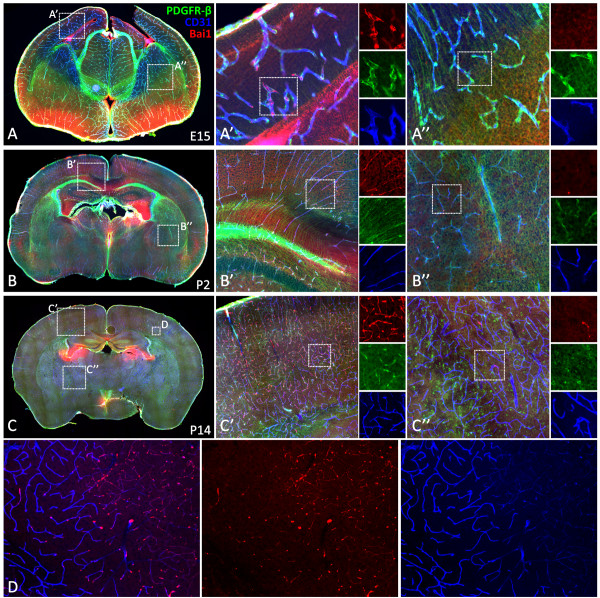
**Bai1 is differentially expressed on growing blood vessels during mice brain development.** Tissue-level characterization of Bai1 expression at E15 (**A**), P2 (**B**) and P14 (**C**, **D**) showed that Bai1 was expressed by cortical blood vessels throughout development (**A’**, **B’** and **C’**). Initially expressed only by cortical vessels (**A’**, **A”**), however, as development progresses, striatal and thalamic vessels also started to express but weakly and non-homogeneously (**A”**, **B”**, and **C”**). Interestingly, at P14, expression was not homogenous in all blood vessels and especially notable on small dense microvessels in the cortex (**D**).

Here, we show that, Bai1 is strongly expressed throughout mouse neurodevelopment at varying levels (Figure 1, Table 2), on average 4- to 5-fold greater than that of Vegf-A receptors, Flt1 and Kdr (Table 2). Our co-immunohistochemistry analysis with endothelial and pericyte cell markers CD31 and PDGFR-beta revealed that, Bai1 is expressed on growing blood vessels of mice brain (Figure 3). Initially around E15, Bai1 is expressed only in the vessels of dorsal cerebral cortex and hippocampus, but not in other brain regions (Figure 3A). At P2, Bai1's expression expands toward the more dorsolateral and ventral cortical areas reaching up to the piriform cortex, but being still absent in subcortical areas (Figure 3B). At P14, Bai1 is strongly expressed in all cortical microvessels as they occupy the whole cortex and to some extent in some of the lateral striatal vessels and dorsal thalamic nuclei, but not in ventral thalamic and hypothalamic vessels at this age (Figure 3C). Notably, expression of the Bai1 was high at the tips of the angiogenic sprouts and branching points, where its function is potentially more needed (Figure 3C’ and D). In those brain parts in which Bai1 is not active, other members of the Bai family, Bai2 and Bai3 might be functioning, especially given the fact that most of the regulatory domains are highly conserved between these members [33].

We also noticed that Bai1 expression is not homogenous throughout the cortex and generally more prominent around dense microvessels at P14 (Figure 3D). This might be because Bai1 function is potentially required especially for endothelial cells found on the angiogenically active small blood vessels to regulate new vessel formation. An alternative reason for this observation might be that the antibody that we used in this study recognizes the N-terminal extracellular domain of the Bai1 (epitope corresponding to amino acids 81–350), which contains a portion of the thrombospondin type-1 repeats found in soluble Vstat40 and Vstat 140 molecules. These thrombospondin type-1 repeats were shown to interact with the CD36 receptor, which is expressed only by endothelial cells on microvessels, but not on larger vessels [33]. Thus, the antibody might be detecting the soluble cleaved product of Bai1 more prominently on small microvessels.

## Conclusion

Expression analysis of angiogenesis-related genes in brain development is a critical initial step to elucidate possible mechanisms and genes that are involved in cerebrovascular pathologies. The data presented here revealed novel angiogenesis-related genes, like Bai1 and Nudt6 that play roles in brain development and in this manner, expected to contribute to basic and clinical research on cerebrovascular diseases.

## Competing interests

The authors declare that they have no competing interests.

## Authors' contributions

AO designed experiments, carried out all experiments and wrote the manuscript. AB contributed to the qPCR experiments. TA contributed to writing the manuscript. AS helped in interpreting the array data. ZOT helped in the statistical calculations. SUB contributed to the immunohistochemistry experiments. ANB helped in design of experiments, supervised the project, critically contributed to preparation and review of the manuscript. TK conceived the ideas, coordinated the project and reviewed the manuscript. All authors read and approved the final manuscript.

## Supplementary Material

Additional file 1 **Table S1.** Normalized expression levels of genes analyzed on the arrays. Click here for file

Additional file 2 **Figure S1.** qPCR expression analysis of Bai1, Nudt6 and Npr1. Experiments repeated three times, error bars shows the standard errors, GAPDH normalized mean values of the three measurements were used. Click here for file
