# Supplementary figures and images for: Temporal expression analysis of angiogenesis-related genes in brain development
Source: Vasc Cell. 2012 Oct 1;4:16. doi: 10.1186/2045-824X-4-16 (PMC3517775; doi:10.1186/2045-824X-4-16)

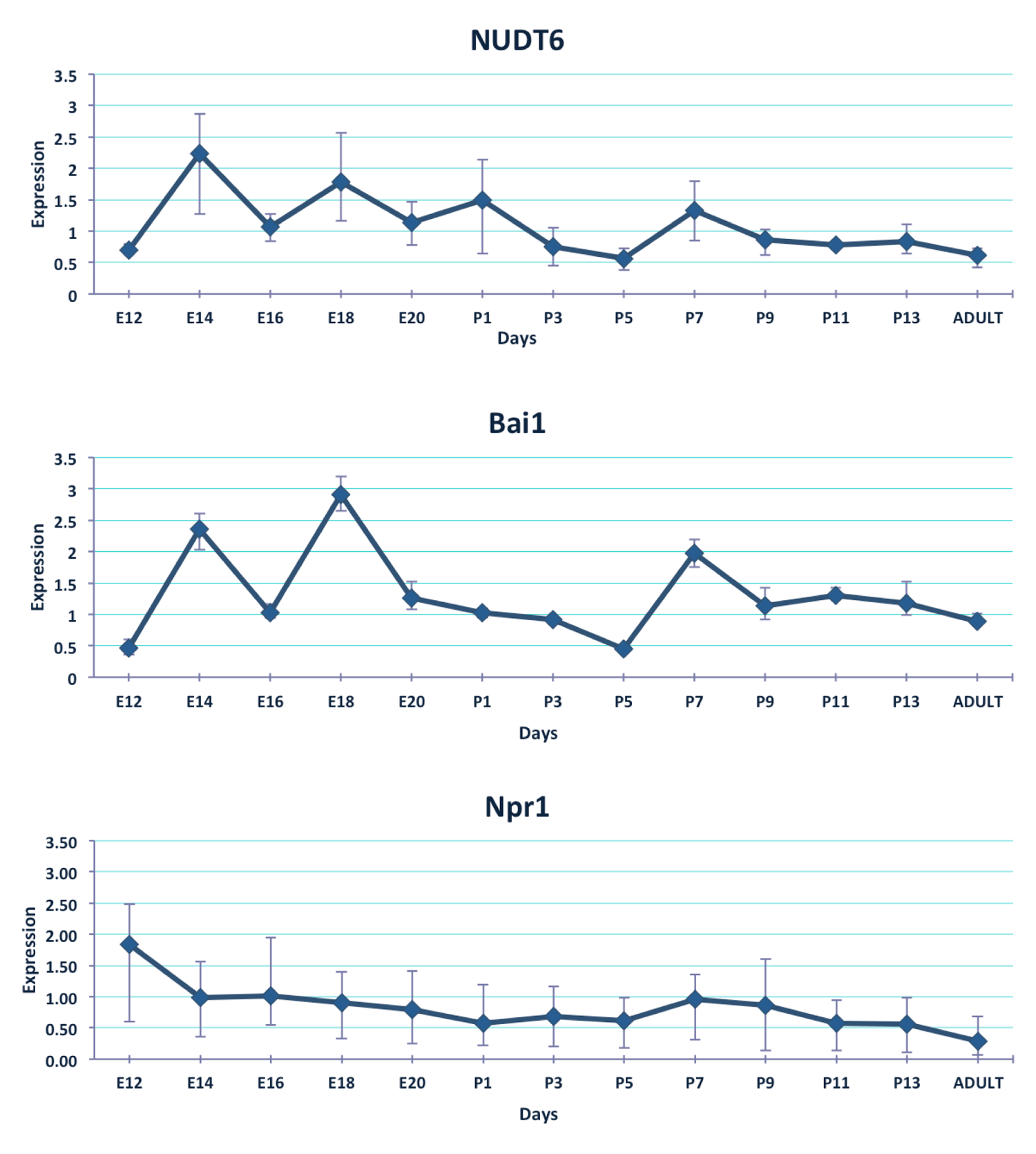

Supplement: Additional file 2 — Figure S1. qPCR expression analysis of Bai1, Nudt6 and Npr1. Experiments repeated three times, error bars shows the standard errors, GAPDH normalized mean values of the three measurements were used. [file 2045-824X-4-16-S2.tiff]
